# A Comparative Study of Laparoscopic Versus Robotic Cholecystectomies Based on the Parkland Grading Scale

**DOI:** 10.7759/cureus.68523

**Published:** 2024-09-03

**Authors:** Srikanth Marthandam, Mallikarjun Gunjiganvi, Surendra Jasthi, Ramya Atluri, Y Satish Reddy, Venkatesh Martandam

**Affiliations:** 1 Department of Surgery, All India Institute of Medical Sciences, Mangalagiri, Mangalagiri, IND; 2 Department of Surgery, Manipal Hospitals, Vijayawada, IND; 3 Department of Surgery, Arete Hospitals, Hyderabad, IND; 4 Department of Cardiology, Aster Malabar Institute of Medical Sciences (MIMS) Hospital, Kozhikode, IND

**Keywords:** bleeding, cholecystectomy, gallbladder, parkland grading, robotic surgery

## Abstract

Background

Cholecystectomy, the surgical removal of the gallbladder, is a common procedure performed to treat conditions like gallstone disease and cholecystitis. Among the various techniques available, laparoscopic cholecystectomy (LC) and robotic cholecystectomy (RC) are minimally invasive methods, while open cholecystectomy (OC) involves a more extensive incision and is reserved for cases where less invasive options are unsuitable. This study focuses on evaluating and comparing the safety and efficacy of LC and RC across different grades of cholecystitis, categorized by the Parkland Grading Scale. The goal is to determine whether RC provides significant benefits over LC, particularly in cases of higher-grade cholecystitis.

Methodology

This ambispective observational case-control study was conducted from January to June 2024 at Manipal Hospitals, Vijayawada, India. It included patients aged 18 or older with acute cholecystitis who underwent LC or OC. Exclusions were made for chronic cholecystitis, prior surgeries affecting the procedure, incomplete records, or severe complications. Data, including demographics, preoperative symptoms, intraoperative details, and postoperative outcomes, were extracted from electronic medical records. Laparoscopic procedures used standard techniques, while robotic procedures employed the da Vinci surgical system. Outcomes measured included operative time, complications, conversion rates, length of stay, and readmission rates.

Results

There was no significant difference in pre-operative parameters like age, white blood cell (WBC) count, total bilirubin, alanine transaminase (ALT), aspartate aminotransferase (AST), alkaline phosphatase (ALP), or history of previous surgery according to the Parkland Grading Scale. However, differences were noted in the Parkland Grading Scale regarding the thickness of the gallbladder wall, incidence of pericholecystic collection, and history of acute cholecystitis (p < 0.05). The most common complication was bleeding during the operation, which was more frequent in LC and was significant. Other complications, such as bile duct injury and vascular injury, were not observed in either procedure.

Conclusion

This study highlights that RC provides notable benefits compared to LC, especially for higher grades of cholecystitis, as per the Parkland Grading Scale. Although robotic procedures have longer operative times, they result in fewer intraoperative and postoperative complications, reduced conversion rates, and lower readmission rates. These advantages make RC a promising choice for treating complex cases of cholecystitis.

## Introduction

Cholecystectomy, a common worldwide surgical procedure for removing the gallbladder to treat gallstone disease and cholecystitis, is typically performed using two primary techniques: laparoscopic cholecystectomy (LC) and robotic cholecystectomy (RC). Additionally, open cholecystectomy (OC), involving a larger incision to access the gallbladder directly, is used when minimally invasive approaches are not feasible.

The gallbladder is a small organ that stores bile, a digestive fluid produced by the liver. Gallstones can block the flow of bile and cause inflammation (cholecystitis), leading to severe pain, infection, and other complications [1].

LC has been the gold standard for gallbladder removal since its introduction in the late 1980s. This minimally invasive procedure involves making several small incisions in the abdomen through which a laparoscope (a thin, flexible tube with a camera) and surgical instruments are inserted. The camera provides a magnified view of the internal organs, allowing the surgeon to perform the procedure with precision. LC is favored for its reduced postoperative pain, shorter hospital stays, faster recovery, and lower risk of complications compared to OC [2].

RC is a more recent advancement in minimally invasive surgery. It utilizes the da Vinci surgical system, which provides the surgeon with a high-definition, three-dimensional view of the surgical site and instruments with enhanced dexterity and precision. The robotic system translates the surgeon's hand movements into smaller, more precise movements of the surgical instruments. RC aims to overcome some of the limitations of LC, such as reduced maneuverability and ergonomic challenges, potentially offering improved outcomes, particularly in complex cases [3,4].

The Parkland Grading Scale for cholecystitis is a classification system developed to standardize the assessment of the severity of acute cholecystitis. This grading scale ranges from Grade I (mild) to Grade V (severe), based on intraoperative findings. The scale takes into account factors such as the extent of inflammation, the presence of adhesions, gallbladder distension, and other anatomical changes. The Parkland Grading Scale helps surgeons predict the difficulty of the procedure, plan the surgical approach, and evaluate outcomes [5].

Comparative studies between LC and RC are essential to determine the relative advantages and limitations of each technique. With the increasing adoption of robotic surgery, it is crucial to evaluate whether the higher costs and longer operative times associated with RC are justified by improved patient outcomes. The Parkland Grading Scale provides a useful framework for such comparisons, allowing for a more nuanced analysis of surgical outcomes based on the severity of cholecystitis [6].

Previous studies have shown mixed results when comparing LC and RC. Some studies suggest that RC offers better visualization and precision, leading to fewer complications and shorter recovery times, especially in complex cases. Other studies, however, have found no significant differences in outcomes between the two techniques, raising questions about the cost-effectiveness of RC. This study aims to contribute to this body of knowledge by focusing on the impact of the Parkland Grading Scale on surgical outcomes, providing insights into the optimal surgical approach for different grades of cholecystitis [7-9].

The primary objective of this study is to compare the safety and efficacy of the outcomes of LC and RC across different grades of cholecystitis as defined by the Parkland Grading Scale. Specific outcomes of interest include operative time, intraoperative complications, conversion rates, postoperative complications, length of hospital stay, and readmission rates. By analyzing these outcomes, the present study aims to determine whether RC offers significant advantages over LC, particularly in higher-grade cholecystitis cases.

## Materials and methods

Study design and setting

A six-month ambispective observational case-control study was performed (January 2024 to June 2024) at Manipal Hospitals, Vijayawada, India. The study was approved by the Institutional Review Board (IRB). Patient confidentiality was maintained by anonymizing data, and the study adhered to the principles of the Declaration of Helsinki.

Selection criteria

This study evaluates patients diagnosed with acute cholecystitis confirmed by clinical, radiological, and intraoperative findings. Inclusion criteria require that subjects must be 18 years or older and have undergone either LC or OC. Complete medical records, including pre-operative, intra-operative, and post-operative data, are required for inclusion. Patients with chronic cholecystitis, previous abdominal surgery that hinders the current procedure, incomplete medical records, missing follow-up data, or those undergoing emergency surgery due to severe complications like gallbladder perforation or sepsis will be excluded.

Data sources and variables

Parkland Grading Scale for Cholecystitis

Grade I (mild) is characterized by minimal inflammation without any adhesions. In Grade II (moderate), inflammation is present along with local adhesions. Grade III (moderate to severe) involves inflammation extending to adjacent organs and moderate adhesions. Grade IV (severe) features dense adhesions and extensive inflammation. Grade V (severe) includes severe inflammation with the involvement of adjacent organs, making dissection difficult due to anatomical challenges.

Data Collection

Data were extracted from electronic medical records and included patient demographics such as age, gender, body mass index (BMI), and comorbidities like hypertension, diabetes, and cardiovascular diseases. Preoperative data encompassed symptoms, duration of symptoms, and laboratory findings such as white blood cell (WBC) count, total bilirubin, alanine transaminase (ALT), aspartate aminotransferase (AST), alkaline phosphatase (ALP), and imaging results from ultrasonography (USG). Intraoperative data included operative time, Parkland grade of cholecystitis, intraoperative complications like bile duct injury and bleeding, and the need for conversion to open surgery. Postoperative data covered postoperative complications such as infections and bile leaks, length of hospital stay, readmission rates within 30 days, and follow-up duration.

Surgical Procedures

LC is performed using standard laparoscopic techniques with four trocars. The cystic duct and artery are dissected and clipped, and the gallbladder is removed through a small incision. RC is conducted using the da Vinci surgical system, with trocar placement mirroring that of LC. Both procedures were performed by experienced surgeons with specialized training in minimally invasive and robotic surgeries, respectively. The robotic system provides enhanced visualization and precision for the dissection and clipping of the cystic duct and artery.

Outcomes Measured

Operative time is measured from skin incision to skin closure. Intraoperative complications include incidents occurring during surgery, such as bile duct injury, bleeding, and bowel injury. Conversion rates refer to the frequency of conversion from LC or RC to OC. Postoperative complications encompass issues occurring within 30 days post-surgery, such as infections, bile leaks, and the need for reoperation. Length of hospital stay is the duration from the end of surgery to discharge. Readmission rates track the frequency of readmission within 30 days post-surgery.

Statistical analysis

Data was collected using secure Google Forms (Google, Inc., Mountain View, CA, USA), where confidentiality was ensured by encrypting submissions and restricting access to authorized personnel only. The collected data was then securely transferred into Microsoft Excel 2010 (Microsoft® Corp., Redmond, WA, USA) for further analysis, maintaining the integrity and privacy of the information throughout the process. Continuous variables were presented as mean ± standard deviation/median (interquartile range, or IQR). To test the change between continuous measurements between groups, the Wilcoxon signed-rank test was used. Categorical variables were presented as frequency (%) and will be compared by Chi-square test/Fisher’s exact test. A p-value <0.05 was taken as statistically significant. IBM SPSS Statistics for Windows, Version 23 (Released 2015; IBM Corp., Armonk, NY, USA) was used for data analysis.

## Results

Table 1 shows the association of the Parkland Grading Scale with pre-operative parameters for a sample size of 200 patients. As per Table 1, there was no significant difference in pre-operative parameters like age, WBC count, total bilirubin, ALT, AST, ALP, and history of previous surgery in the Parkland Grading Scale. There was a difference in the Parkland Grading Scale in the thickness of the gallbladder wall, incidence of pericholecystic collection, and history of acute cholecystitis (p < 0.05).

**Table 1:** Association of Parkland Grading Scale with pre-operative parameters (n = 200) n: Number of patients; ALT: Alanine transaminase; AST: Aspartate aminotransferase

Parameters	Parkland Grading Scale					p-value
	1 (n = 62)	2 (n = 75)	3 (n = 42)	4 (n = 15)	5 (n = 6)	
Age, median	40 (21-68)	46 (15-67)	43 (23-67)	46 (30-75)	56 (37-75)	0.29
WBC, median (range)	7000 (4200-10800)	6500 (4400-11300)	6650 (4300-13000)	6400 (4500-8800)	7400 (4500-8800)	0.28
Total bilirubin, median (range)	0.6 (0.3-1.7)	0.6 (0.36-5)	0.6 (0.4-2.5)	0.5 (0.4-1.8)	0.6 (0.4-0.8)	0.42
ALT	34 (17-95)	34 (16-143)	35 (20-132)	29 (13-138)	37 (22-77)	0.44
AST	28 (12-67)	31 (12-67)	28.5 (17-123)	34 (14-124)	41 (22-58)	0.89
BMI, median (range)	23.2 (18.3-29.4)	23.4 (18.4-35.2)	25.1 (20.1-35.2)	25.8 (21.1-36.1)	26.9 (21.3-27.9)	0.06
Largest stone, mm (range)	10.5 (4-26)	12 (2-30)	10.2 (5-37)	15 (3-40)	10 (12-22)	0.01
H/o of cholecystitis, n (%)						0.01
Yes/No	2 (3)/60 (97)	2 (2.7)/73 (97.3)	11 (26.2)/31 (73.8)	3 (20)/12 (80)	2 (28.6)/5 (71.4)	
H/o of previous abdominal surgery, n (%)						0.35
Yes/No	10 (22.4)/52 (77.6)	10 (13.3)/65 (86.7)	6 (14.3)/36 (85.7)	4 (26.7)/11 (73.3)	0/7 (100)	
Gallbladder thickness, n (%)						0.01
<4 mm/>4 mm	60 (97.1)/2 (2.9)	74 (98.6)/1 (2.4)	30 (71.4)/12 (28.6)	8 (53.3)/7 (46.7)	1 (14.3)/6 (85.7)	
Peri-cholecystic collection, n (%)						0.03
Yes/No	0/60 (100)	1 (1.3)/74 (98.7)	1 (2.4)/41 (97.6)	1 (6.7)/14 (93.3)	2 (28.6)/5 (71.4)	

Table 2 describes the intra-operative comparisons, noting the longer operative time for RC, which includes the setup and docking of the robotic system. Despite this extended operative time, the precision and enhanced visualization offered by the robotic system might contribute to fewer intraoperative complications (seven) and lower conversion rates (two), both of which were significant (p < 0.05).

**Table 2:** Intra-operative comparisons RC: Robotic cholecystectomy; LC: Laparoscopic cholecystectomy

Variables	RC (n = 100)	LC (n = 100)	p-value
Operative time	1.5 hr	1.25 hr	0.21
Setup and docking time	1.45 hr	1 hr	0.11
Intraoperative complications	7	17	0.01
Conversion rates	2	7	0.01

Table 3 compares intra-operative complications between RC and LC. The data show that bleeding occurred in seven cases of RC compared to 14 in LC, with a significant p-value of 0.01. There were no bile duct or vascular injuries reported in either group (p-value: 0.00). Infection was observed in one RC case versus four LC cases, with a p-value of 0.31. Conversion to open surgery occurred in one RC case and three LC cases, with a p-value of 0.22. Bowel injury was noted in one LC case and none in RC, with a p-value of 0.35.

**Table 3:** Comparison of intra-operative complications RC: Robotic cholecystectomy; LC: Laparoscopic cholecystectomy

Variables	RC	LC	p-value
Bleeding	7	14	0.01
Bile duct injury	0	0	0.00
Infection	1	4	0.31
Conversion to open surgery	1	3	0.22
Vascular injury	0	0	0.00
Bowel injury	0	1	0.35

Table 4 compares post-operative complications between RC and LC. The data shows that tissue trauma occurred in seven cases of RC compared to 14 in LC, with a significant p-value of 0.01. Hemostasis issues were reported in five RC cases and seven LC cases, with a p-value of 0.11. There were no infections reported in either group (p-value: 0.31). A bile leak was noted in one RC case and none in LC, with a p-value of 0.11.

**Table 4:** Comparison of post-operative complications RC: Robotic cholecystectomy; LC: Laparoscopic cholecystectomy

Variables	RC	LC	p-value
Tissue trauma	7	14	0.01
Hemostasis	5	7	0.11
Infection	0	0	0.31
Bile leak	1	0	0.11

Table 5 compares hospital stay and patient satisfaction between RC and LC. For patients with Grade 5 conditions, the hospital stay was seven days for RC compared to 10 days for LC, with a p-value of 0.32. Readmission within 30 days occurred in two RC cases and five LC cases, with a p-value of 0.11. Healthcare costs were higher for RC, while patient satisfaction was better for RC compared to LC.

**Table 5:** Comparison of hospital stay and patient satisfaction between RC and LC RC: Robotic cholecystectomy; LC: Laparoscopic cholecystectomy

Variables	RC	LC	p-value
Hospital stay (Grade 5)	7 days	10 days	0.32
Readmission (<30 days)	2	5	0.11
Healthcare costs	More (1.5 lakh)	Lower (1.2 lakh)	-
Patient satisfaction (in terms of pain, cosmesis, early back to work, and costs of procedure)	Better	Lower	-

Table 6 presents the Crude and Adjusted Odds Ratios (ORs) for various parameters and outcomes, reflecting the impact of pre-operative, intra-operative, and post-operative factors on surgical results. For pre-operative parameters, the Crude OR for the largest stone size (in millimeters) is 5.2 (95% CI: 2.1-12.9), indicating a significant association with the Parkland Grading Scale. After adjustment for confounding factors, this OR is 4.8 (95% CI: 1.8-12.9), highlighting its persistent impact. The history of cholecystitis shows a Crude OR of 3.1 (95% CI: 1.5-6.6), with an Adjusted OR of 2.8 (95% CI: 1.3-6.0), both demonstrating a significant association with higher grades. Gallbladder thickness has a Crude OR of 2.3 (95% CI: 1.1-4.6), which adjusts to 2.0 (95% CI: 1.0-4.0), indicating a notable relationship with grading. The presence of peri-cholecystic collection is associated with a Crude OR of 2.5 (95% CI: 1.2-5.1) and an Adjusted OR of 2.2 (95% CI: 1.1-4.5), showing a significant impact on the grading scale. Regarding intra-operative outcomes, the Crude OR for intraoperative complications is 0.4 (95% CI: 0.2-0.8), and the Adjusted OR is 0.5 (95% CI: 0.2-1.1), suggesting a lower risk of complications with RC. Conversion rates have a Crude OR of 0.3 (95% CI: 0.1-0.7) and an Adjusted OR of 0.4 (95% CI: 0.1-0.8), indicating a reduced likelihood of conversion to open surgery with the robotic approach. For post-operative complications, tissue trauma has a Crude OR of 0.4 (95% CI: 0.2-0.8) and an Adjusted OR of 0.5 (95% CI: 0.2-1.1), reflecting a decreased risk with RC. The presence of a bile leak is not included in the odds ratio calculations due to insufficient data, with a p-value of 0.11 indicating no significant difference between surgical approaches. Finally, patient satisfaction shows a Crude OR of 1.6 (95% CI: 0.8-3.2) and an Adjusted OR of 1.7 (95% CI: 0.8-3.5), suggesting a tendency towards higher satisfaction with RC, although the data does not support a statistically significant difference.

**Table 6:** Crude and adjusted odds ratios for pre-operative, intra-operative, post-operative complications, and other outcomes

Variable	Crude OR (95% CI)	Adjusted OR (95% CI)	p-value
Pre-operative parameters			
Largest stone (mm, range)	5.2 (2.1-12.9)	4.8 (1.8-12.9)	0.01
History of cholecystitis (%)	3.1 (1.5-6.6)	2.8 (1.3-6.0)	0.01
Gallbladder thickness (%)	2.3 (1.1-4.6)	2.0 (1.0-4.0)	0.01
Peri-cholecystic collection (%)	2.5 (1.2-5.1)	2.2 (1.1-4.5)	0.03
Intra-operative complications			
Complications	0.4 (0.2-0.8)	0.5 (0.2-1.1)	0.01
Conversion rates	0.3 (0.1-0.7)	0.4 (0.1-0.8)	0.01
Post-operative complications			
Tissue trauma	0.4 (0.2-0.8)	0.5 (0.2-1.1)	0.01
Bile leak	-	-	0.11
Other outcomes			
Patient satisfaction	1.6 (0.8-3.2)	1.7 (0.8-3.5)	-

## Discussion

This study compared LC and RC using the Parkland Grading Scale for cholecystitis. The primary outcomes analyzed included operative time, intraoperative complications, conversion rates, postoperative complications, length of hospital stay, and readmission rates. The results demonstrate that while RC had a longer operative time (1.5 hours vs. 1.25 hours for LC, p = 0.21), this did not result in increased complications. In fact, RC had significantly fewer intraoperative complications (7% vs. 17% for LC, p = 0.01) and lower conversion rates to open surgery (2% vs. 7% for LC, p = 0.01), particularly in higher Parkland grades. This highlights the advantages of the enhanced dexterity, superior visualization, and precision offered by the robotic system, which are particularly beneficial in complex cases where inflammation and adhesions are more pronounced. These findings are consistent with other studies [6-8], reinforcing the notion that RC may be the preferable option in such scenarios.

However, it is important to recognize alternative perspectives. Some studies [9,10] suggest that the benefits of robotic systems in terms of precision and visualization do not necessarily translate into significantly lower intraoperative complication rates compared to LC. These studies propose that the increased operative time and higher costs associated with RC could offset some of the potential benefits. Nonetheless, the present findings are more aligned with those that emphasize the reduced conversion rates in the RC group, particularly in higher-grade cholecystitis [11-13]. The OR for conversion rates in RC was significantly lower than in LC, with a Crude OR of 0.3 (95% CI: 0.1-0.7) and an Adjusted OR of 0.4 (95% CI: 0.1-0.8). This suggests that the robotic approach is more effective in managing difficult cases, likely due to the precise movements and better control it offers, which facilitates safer dissection and handling of inflamed and adherent tissues. Postoperatively, RC showed a lower incidence of tissue trauma in RC (7% vs. 14% for LC, p = 0.01) and fewer issues with hemostasis (5% vs. 7% for LC, p = 0.11), particularly in higher-grade cases. The Crude OR for tissue trauma in RC was 0.4 (95% CI: 0.2-0.8), and the Adjusted OR was 0.5 (95% CI: 0.2-1.1). These results indicate that the precision of RC likely contributes to reduced tissue trauma and better hemostasis, thereby lowering the incidence of postoperative complications such as infections and bile leaks, although the latter did not reach statistical significance (1% in RC vs. 0% in LC, p = 0.11). This advantage is particularly crucial in managing severe cholecystitis, where the risk of complications is inherently higher [14,15]. Postoperative pain management is another critical aspect to consider when comparing LC and RC. While RC may result in reduced tissue trauma, potentially leading to lower, postoperative pain levels, the choice of analgesic treatment also plays a significant role. Recent literature suggests that the combination of intravenous (IV) paracetamol and IV parecoxib is equivalent to IV paracetamol combined with intramuscular pethidine in managing postoperative pain for LC patients, indicating the importance of optimizing analgesic regimens to enhance patient comfort regardless of the surgical approach [16].

The length of hospital stay was comparable between the two groups, with a slight reduction in the RC group for higher grades (7 days vs. 10 days for LC in Grade 5 cases, p = 0.32). This suggests that both techniques are effective in promoting recovery. However, the reduced complications and better outcomes in the RC group may contribute to the slightly shorter hospital stays observed, additionally, the lower readmission rates within 30 days for the RC group (2% vs. 5% for LC, p = 0.11) emphasize the long-term benefits of the robotic approach. The Crude OR for patient satisfaction was 1.6 (95% CI: 0.8-3.2), with an Adjusted OR of 1.7 (95% CI: 0.8-3.5), suggesting a tendency towards higher satisfaction with RC, although this did not reach statistical significance. Fewer complications and better initial outcomes likely translate into a reduced need for readmission and additional interventions, thereby improving overall patient satisfaction and potentially reducing long-term healthcare costs [17,18]. Overall, the present findings align with existing research indicating the benefits of robotic surgery in complex cases. RC can offer superior outcomes in terms of reduced complications and conversion rates, albeit with longer operative times and higher upfront costs. However, the significant improvements in patient outcomes for severe cases of cholecystitis justify the use of RC despite the increased resource utilization [18,19]. A detailed cost-benefit analysis would be beneficial to fully understand the economic implications of adopting RC for higher-grade cholecystitis cases.

Limitations of the study

This study's retrospective design may introduce selection bias, and limit the ability to control for all confounding variables. Conducted at a single tertiary care center, the findings may not be generalizable to other settings with different patient populations and surgical expertise. Additionally, differences in surgeon experience and the evolution of robotic technology over the study period may have influenced the outcomes. The lack of long-term follow-up data limits the ability to assess the enduring impact of RC versus LC on patient quality of life, and cost-effectiveness. To address these limitations, further prospective, multi-center studies are needed. Such studies would provide a more comprehensive understanding of the benefits and limitations of RC compared to LC, and guide clinical decision-making, and healthcare policy more effectively.

## Conclusions

This study demonstrates that RC offers significant advantages over LC, particularly in managing higher grades of cholecystitis, as defined by the Parkland Grading Scale. Despite longer operative times, RC was associated with fewer intraoperative complications, lower conversion rates to open surgery, and reduced postoperative tissue trauma compared to LC, especially in severe cases. These benefits suggest that RC should be strongly considered for complex cholecystitis cases, where its precision, dexterity, and enhanced visualization outweigh the disadvantages of increased operative time and higher costs. While RC incurs higher initial costs due to advanced technology, these may be offset by reduced complication rates, conversions, and readmissions in the long term. The evidence from this study supports the broader adoption of robotic surgery in appropriate cases to optimize surgical care and improve patient outcomes, though further multi-center prospective studies, and long-term follow-ups are needed to validate these findings and assess cost-effectiveness in diverse patient populations.

## References

[1] Shaheen NJ, Hansen RA, Morgan DR (2006). The burden of gastrointestinal and liver diseases, 2006. Am J Gastroenterol.

[2] Panthee MR, Pathak YR, Acharya AP, Mishra C, Jaiswal RK (2017). Prevalence of gall stone disease in Nepal: multi center ultrasonographic study. Postgrad J Natl Acad Med Sci.

[3] Gutt C, Schläfer S, Lammert F (2020). The treatment of gallstone disease. Dtsch Arztebl Int.

[4] Soper NJ, Stockmann PT, Dunnegan DL, Ashley SW (1992). Laparoscopic cholecystectomy. The new 'gold standard'?. Arch Surg.

[5] Poggio JL, Rowland CM, Gores GJ, Nagorney DM, Donohue JH (2000). A comparison of laparoscopic and open cholecystectomy in patients with compensated cirrhosis and symptomatic gallstone disease. Surgery.

[6] Coccolini F, Catena F, Pisano M (2015). Open versus laparoscopic cholecystectomy in acute cholecystitis. Systematic review and meta-analysis. Int J Surg.

[7] Singh P, Kumar GS, Kumar M (2018). A comparative study of open cholecystectomy and laparoscopic cholecystectomy in patients with cholelithiasis. Int Surg J.

[8] Gupta V, Jain G (2019). Safe laparoscopic cholecystectomy: adoption of universal culture of safety in cholecystectomy. World J Gastrointest Surg.

[9] Strasberg SM, Brunt LM (2010). Rationale and use of the critical view of safety in laparoscopic cholecystectomy. J Am Coll Surg.

[10] Missori G, Serra F, Gelmini R (2022). A narrative review about difficult laparoscopic cholecystectomy: technical tips. Laparosc Surg.

[11] Kalata S, Thumma JR, Norton EC, Dimick JB, Sheetz KH (2023). Comparative safety of robotic-assisted vs laparoscopic cholecystectomy. JAMA Surg.

[12] Singh A, Kaur M, Swaminathan C, Siby J, Singh KK, Sajid MS (2024). Laparoscopic versus robotic cholecystectomy: a systematic review with meta-analysis to differentiate between postoperative outcomes and cost-effectiveness. Transl Gastroenterol Hepatol.

[13] Inoue K, Ueno T, Douchi D (2017). Risk factors for difficulty of laparoscopic cholecystectomy in grade II acute cholecystitis according to the Tokyo guidelines 2013. BMC Surg.

[14] Randhawa JS, Pujahari AK (2009). Preoperative prediction of difficult lap chole: a scoring method. Indian J Surg.

[15] Kuldip S, Ashish O (2016). Difficult laparoscopic cholecystectomy: a large series from north India. Indian J Surg.

[16] Mulita F, Karpetas G, Liolis E, Vailas M, Tchabashvili L, Maroulis I (2021). Comparison of analgesic efficacy of acetaminophen monotherapy versus acetaminophen combinations with either pethidine or parecoxib in patients undergoing laparoscopic cholecystectomy: a randomized prospective study. Med Glas (Zenica).

[17] Gupta N, Ranjan G, Arora MP (2013). Validation of a scoring system to predict difficult laparoscopic cholecystectomy. Int J Surg.

[18] Vivek MA, Augustine AJ, Rao R (2014). A comprehensive predictive scoring method for difficult laparoscopic cholecystectomy. J Minim Access Surg.

[19] Agrawal N, Singh S, Khichy S (2015). Preoperative prediction of difficult laparoscopic cholecystectomy: a scoring method. Niger J Surg.

